# Pathologic Signaling and Disease Implications of Insulin-like Growth Factor Binding Proteins in Cancer, Cardiovascular Disease, and Fibrosis

**DOI:** 10.3390/ijms262110248

**Published:** 2025-10-22

**Authors:** Zachary R. Sechrist, Jaeden S. Cortés, Nidhi R. Patel, Zoe J. Pittman, Gayathri Guru Murthy, Guangzhen Zhu, Calvin L. Cole, Benjamin D. Korman

**Affiliations:** 1 Department of Pathology and Laboratory Medicine, University of Rochester Medical Center, Rochester, NY 14642, USA; zachary_sechrist@urmc.rochester.edu; 2 Department of Surgical Oncology, University of Rochester Medical Center, Rochester, NY 14642, USA; 3 Center for Musculoskeletal Research, University of Rochester Medical Center, Rochester, NY 14642, USA; 4 Translational Biomedical Science Program, University of Rochester Medical Center, Rochester, NY 14642, USA; 5 Department of Allergy, Immunology & Rheumatology, University of Rochester Medical Center, Rochester, NY 14642, USA

**Keywords:** IGFBP, cancer, cardiovascular disease, fibrosis, IGF, TGF-β, hypoxia, angiogenesis, epithelial-mesenchymal transition (EMT)

## Abstract

The insulin-like growth factor binding protein (IGFBP) family consists of seven members, which are known for their roles in regulating canonical insulin-like growth factor (IGF) signaling and, more recently, a variety of non-canonical signaling pathways. This review will explore shared pathologic mechanisms amongst the IGFBP family members in diseases like cancer and fibrosis which reveal the unique and redundant properties of this critical family of proteins and provides unique insight into how their regulation is not only relevant to cell metabolism, but also plays an emerging role in diverse processes including immunity, TGF-β signaling, hypoxia and angiogenesis, and senescence. Moreover, these molecules have shown promise both as biomarkers and therapeutics, and a deeper understanding of this system is needed to appreciate how leveraging their regulation may be able to ameliorate diverse organ pathologies.

## 1. Introduction

The insulin-like growth factor binding protein (IGFBP) family consists of seven well-characterized members. Traditionally, they are known for their roles in regulating insulin-like growth factors (IGF-1 and IGF-2) and insulin signaling in a variety of tissue types helping to maintain homeostasis. More recently, these proteins have been found to signal through IGF-1R-independent signaling and are associated with a diverse group of signaling pathways which allow these molecules to alter cellular function beyond AKT/mTOR signaling and participate in the pathogenesis of a variety of otherwise seemingly divergent disease states. Biomarker studies have shown clear links between IGFBPs and a variety of pathologies including cancer, fibrosis, and vascular disease, but it is only in the last several years that mechanistic studies have begun to shed light on the role that these molecules play in disease. This review will identify the role that dysregulation of IGFBP family members have on disease progression and highlight conserved signaling mechanisms across independent pathological states. An enhanced understanding of IGFBPs can inform the development of new therapeutics for frequently intractable disease states.

## 2. IGFBP Structure and Function

In eukaryotes, IGF-1 and IGF-2 bind with high affinity to 6 homologous proteins known as IGFBPs. However, the seventh protein of the IGFBP family, IGFBP-7, binds to IGF-1 and IGF-2 with low affinity, and binds to insulin-growth factor 1 receptor (IGF-1R) and insulin more readily [1]. The IGFBPs are synthesized in the liver and are believed to have originated from a single ancestral IGFBP in chordates that then duplicated into two IGFBP genes. Following two genome duplications, eight IGFBP genes remained in placental mammals; however, two were lost, resulting in closely related IGFBPs 1-6 that have been successfully cloned and remain conserved in placental mammals to this day [2]. A total of 15 IGFBPs have since been discovered and categorized based on their affinity for IGFs. IGFBPs 1-6 are categorized as high-affinity binding proteins, whereas IGFBP-related proteins 1-10 (IGFBP-rP 1-10) are categorized as low-affinity binding proteins, with a higher affinity for insulin than to IGFs. Of the 10 IGFBP-related proteins, IGFBP-rp1 was the first discovered and successfully cloned as a gene and was classified as the seventh IGFBP [3,4]. Structurally, IGFBPs 1 through 6 comprise highly conserved N-terminus, central linker, and C-terminus domains unique to each protein. The N-terminus contains an IGFBP motif, which is an essential region for IGF binding, and 16–18 cysteine residues conserved across the IGFBPS that are bonded by intradomain disulfide bonds which contribute to the globular structure and high IGF affinity of these proteins. The C-terminus, while less conserved, comprises only six conserved cysteine residues. The central domain provides further specificity to each of the IGFBPs as it contains glycosylation, phosphorylation, and proteolytic cleavage sites [2,3]. While IGFBP-7 can bind IGFs with low affinity at its N-terminus, it lacks the conserved cysteine residue and the glycosylation and phosphorylation motifs present in the other IGFBPs, explaining its reduced specificity to bind IGF [3].

### 2.1. Canonical Functions and Maintenance of Homeostasis

Canonically, IGFBPs function to bind IGF-1 and 2, increasing the circulatory half-life of IGFs and modulating their binding to their principal receptor, IGF receptor 1 (IGF-1R). IGFBPs inhibit or augment IGF-1R signaling via post-translational modification or interactions with other regulatory proteins. This regulates signal transduction of IGF-1R and other processes including cellular growth, differentiation, and apoptosis [3,5]. Without inhibition of IGF/IGF-1R signaling, the PI3K-AKT/mTOR pathway, responsible for anti-apoptotic effects/protein synthesis, and the MAPK pathway, responsible for mitogenesis, are both activated [6,7]. When IGFs are bound to IGFBP binary complexes, their half-lives are extended by 90 min, up to 20 h for IGFBPs that can form a ternary complex with the acid-labile subunit (ALS). This allows for the accumulation of large reservoirs of IGFs that would otherwise be secreted immediately due to the lack of IGF intracellular tissue stores. IGFBP-7 is the only IGFBP that binds insulin, with a 100-fold lower affinity for IGF-1 and -2 [3].

IGF-1R signaling is not only inhibited through high-affinity binding of the IGFBPs to IGFs at the cell surface, but also by way of other mechanisms including phosphorylation. IGFBP-1, for example, has an increased affinity for IGF-1 due to hyperphosphorylation of its serine residues. This in turn reduces phosphorylation of IGF-1R and further reduces IGF-1R activation by IGF-1 [5]. IGFBP-1 has also been found to modulate IGF-1 availability metabolically. The production of IGFBP-1 in the liver is inversely related to insulin production, whereby insulin suppresses IGFBP-1 production. In a fasted state, as insulin levels decrease, there is a release of IGFBP-1 from the liver which in turn reduces IGF activity [2].

IGFBP-3 is the most abundant circulatory protein of the seven IGFBPs. IGFBP-3 enables endocrine signaling of IGFs by enhancing the stability of the protein in circulation. This allows IGF to reach distal tissue and induce IGF-1R signaling. The C-terminus of IGFBP-3, when bound to IGFs in a binary complex between 30 and 40 kDa, can bind the glycoprotein ALS to form a ternary complex of approximately 150 kDa. This complex is too large to migrate through vascular endothelium, resulting in increased circulatory IGF concentration [2,8]. Although approximately 90% of IGF:ALS ternary structures are assembled with IGFBP-3, IGFBP-5 is also capable of interacting with IGFs and ALS in circulation, with 50% of IGFBP-5 existing within the IGF:ALS ternary complex [2,5]. Proteolysis of the central linker domain of IGFBPs 3 and 5 results in the release of IGF from the ternary complexes, which increases the available concentration of biologically active IGF [9].

Proteolysis is not limited to IGFBP-3 and -5, as it is a mechanism through which all IGFBPs potentiate IGF-1R signaling by weakening IGF-IGFBP interactions. IGF signal transduction is initiated when IGFBP linker domains or C-terminals are cleaved by proteases, including matrix metalloproteases, kallikreins, cathepsins, and complement component C1. Recent studies have shown that pappalysin metalloproteinase PPAP-A is able to cleave IGFBPs 2, 4, and 5, whereas PPAP-A2 has been shown to cleave IGFBPs 3 and 5 [5,8,10].

The termination of cell replication, known as cellular senescence, is a regulatory mechanism induced in aged or stressed cells that can maintain homeostasis or be pathologic depending on cellular context, as often mobilized as an anti-cancer mechanism. IGFBPs 1-7 are part of the senescence-associated secretory phenotype (SASP), which constitutes proteins that are secreted by senescent cells. IGFBPs 4, 5, and 7 participate in a positive feedback loop in which senescent cells secrete reactive oxygen species (ROS), resulting in the secretion of IGFBPs which then induce senescence in neighboring cells via paracrine signaling [1,11]. Prolonged IGF activity has also been linked to promoting senescent phenotypes within cells, and IGFBP binding to either IGFs or IGFRs can modulate the development of cellular senescence. IGFBP-4’s recruitment of PAPPA tends to increase senescence, while IGFBP-3 tends to increase circulatory IGF levels, inhibiting IGF activity [10]. While IGFBP-7 exhibits relatively weak binding affinity to IGFs, it can bind IGF-1R, which is a binding partner of IGF-2. By competitively binding this receptor, IGF-2 is redirected to IGF-2R, which then promotes senescence. IGF-2-induced senescence via IGFBP-5 and 7 has also been shown to utilize the ERK and AKT signaling pathways [1,12].

Interestingly, by regulating the bioavailability of their canonical binding partners IGF-1 and -2, IGFBPs play a predominant role in folliculogenesis and embryogenesis [13]. IGFBP-2 is found to be expressed in highly proliferating cells and rapidly developing tissues during embryogenesis and enhances tissue development [14]. Similarly, IGFBP-3 is expressed by cells of the maternal reproductive tract and the embryo during pre-implantation, and has been shown to increase the developmental rate of embryos both in vivo and in vitro through IGF-1R signaling [15]. Importantly, disruptions to IGFBP signaling during embryogenesis can lead to impaired development. Elevated or phosphorylated IGFBP-1 leads to sequestration of IGF-1 and inhibits IGF-1R signaling, resulting in fetal growth restriction [16,17]. Lastly, IGFBP-4, a known inhibitor of the IGFs, has been observed to decrease in concentration in dominant bovine ovarian follicles and was upregulated in follicle atresia. Recombinant bovine IGFBP-4 significantly reduces hatching initiation and completion rates of blastocytes at the highest concentration [18]. Together, this highlights the importance of the IGFBP family of proteins in development and homeostatic functions. Better understanding the temporal regulation and intricate signaling of IGFBPs during embryogenesis can further identify their role in conditions like cancer, fibrosis and vascular disease.

### 2.2. Non-Canonical Functions and Pathological Pathways

While the canonical processes represent well-established functions of IGFBPs, this family of proteins also exhibits non-canonical functions that are independent of IGF activity such as integrin binding and nuclear translocation. Integrins are transmembrane proteins that allow for cell adhesion and communication between extracellular matrix proteins and cytoskeleton. Through non-canonical IGFBP binding, integrin activation modulates several cellular processes such as expression of genes, and ultimately tumorigenesis and disease outcomes. IGFBPs 1 and 2 contain the widely recognized integrin-binding tripeptide Arg-Gly-Asp (RGD), enabling them to bind to cell surfaces and modulate a number of cellular processes by inducing kinase phosphorylation [2,5]. IGFBP-1 has been shown to carry out cell-specific IGF-independent functions influencing cell motility via the RGD motif binding to alpha5- β1 integrin. While this complex enhances cell migration in cell types such as Chinese hamster ovary, smooth muscle, and schwannoma cells, cell invasion is inhibited in cell types such as mammary tumor cells and endometrial stromal cultures. Similarly, IGFBP-2 promotes tumorigenesis and enhances cell proliferation via integrin β1 activation and associated signaling in many cell types. Glioma cell proliferation that was promoted by exogenous IGFBP-2-induced integrin/ERK activation was inhibited by anti-integrin β1 antibody as well as knockdown of the protein [19]. IGFBP-2 also induces vasculogenic mimicry formation in glioma tissue via integrin alpha-5 and β1/RGD binding as adhesion proteins expressed in endothelial cells such as CD144 are upregulated [20]. Hepatic Stellate Cell (HSC) migration, a marker of liver fibrosis, is also promoted through IGFBP-induced integrin β1 signaling, more specifically integrin-β1-induced AKT phosphorylation. IGFBP-3 treated HSCs that are incubated with integrin β1-neutralizing antibody experience blocked cellular migration and attenuated AKT phosphorylation [21].

Nuclear translocation, another non-canonical action of IGFBPs, is the process by which cargo proteins are transported across the nuclear membrane, where they interact with nuclear hormones or receptors to modulate transcription of target genes. Select IGFBPs have been observed to participate in non-canonical signaling via nuclear translocation, in which they influence transcriptional activity by most commonly binding to the nuclear receptor retinoid X receptor alpha (RXR-α). RXR-α forms dimeric structures with nuclear receptors including retinoic acid receptor (RAR), peroxisome proliferator-activated receptor-λ (PPAR-λ), vitamin D receptor (VDR), and Nur77 [2]. IGFBP-3 contains a nuclear localization sequence (NLS) at the C-terminal that binds importin-β, a receptor protein responsible for transporting cargo proteins in and out of the nucleus via the nuclear pore complex (NPC) [2]. The N and C terminals of IGFBP-3 are then able to interact with nuclear receptors RXR-α or RAR existing as homodimers or heterodimers with their affiliated binding partners, whose response elements activate or inhibit transcription, respectively. Nur77, when associated with IGFBP-3 in a ternary complex, is phosphorylated and exported from the nucleus. This transport results in cytochrome c release, alongside caspase 3 and 7 activation, inducing apoptosis [2,22]. Like IGFBP-3, IGFBP-5 contains an NLS within its C-terminal that facilitates its nuclear translocation through interacting with importin α/β complexes and nucleon. IGFBP-5 has been shown to bind nuclear receptor VDR, preventing RXR/VDR heterodimerization and therefore attenuating the expression of bone differentiation markers in osteoblast-like cells [5,23]. IGFBP-5 has also been observed to bind RAR and interact with the natural RAR ligand all-trans retinoic acid (ARTA), impacting transcriptional activity associated with senescence [24]. IGFBP-6 is similarly translocated into the nucleus via an NLS located on the C-terminus, exhibiting preferential binding to importin-α. IGFBP-6 competitively binds to RXR-binding partners VDR and TRα1, preventing heterodimerization and thus attenuating VDR-dependent and TRα1-dependent transcriptional activity [5]. IGFBP-2 differs from the other IGFBPs capable of nuclear translocation in the sense that its NLS is located within the linker domain, which interacts with importin-α. Instead of binding nuclear receptors, IGFBP-2 is known to interact with the Vascular Endothelial Growth Factor (VEGF) promoter via transcription factor Fra-1, upregulating VEGF transcription and promoting angiogenesis [5].

While senescence is a normal cellular process, it can also occur non-canonically and promote tumorigenesis and disease progression. For example, mesenchymal stem cells (MSCs) treated with IGFBP-4 demonstrate an increase in SA-beta-gal, a biomarker for senescence [5]. Additionally, IGFBP-4 potentiates IGF-2’s induction of senescence as IGF-2 degradation is delayed in the presence of IGFBP-4 [11]. IGFBP-5 is also a promoter of senescence in human dermal fibroblasts (HDFs). Y-27632 is a compound that inhibits Rho-kinases that function to promote epidermal cell growth via ROCK 1 and ROCK 2. HDFs treated with y-27632 acquire a senescence-associated secretory phenotype (SASP), converting them to cancer-associated fibroblasts (CAF) which can be inhibited by IGFBP-5 knockdown [25]. Like IGFBP-4 and -5, IGFBP-7 can induce senescence in MSCs through release of SASP factors as well as through insulin and IGF interaction. When healthy MSCs were incubated with IGFBP-7, senescence was promoted. Additionally, unlike the other IGFBPs, IGFBP-7 can induce senescence in MSCs by binding insulin and blocking its anti-senescent properties [1]. IGFBP-7-induced senescence is exhibited in the progression of diseases such as scleroderma, in which human dermal fibroblasts cultured with both IGFBP-7 and TGF-β1 upregulated the expression of genes associated with myofibroblast differentiation and cellular senescence compared to healthy control fibroblasts [26].

In recent years, dysregulated canonical and non-canonical signaling of IGFBPs has gained interest for its role in disease states like cancer, vascular disease and fibrosis. Despite a well-established definition of IGFBP structure, pathological signaling is poorly understood and displays cell- and context-dependent differences in the literature. Interestingly, IGFBP family members display shared, complex roles in regulating the immune system, non-canonical TGF-β signaling, epithelial-to-mesenchymal transition, matrix remodeling, hypoxia, and angiogenesis. Importantly, dysregulation in these mechanisms is instrumental in the development of various cancers, vascular disease and fibrosis, which remain poorly treated diseases associated with poor patient quality of life. Understanding the dependence of IGFBP signaling and downstream mechanisms in disease may lead to improved clinical therapies.

## 3. IGFBP Signaling Pathways in Cancer

**Figure 1:**
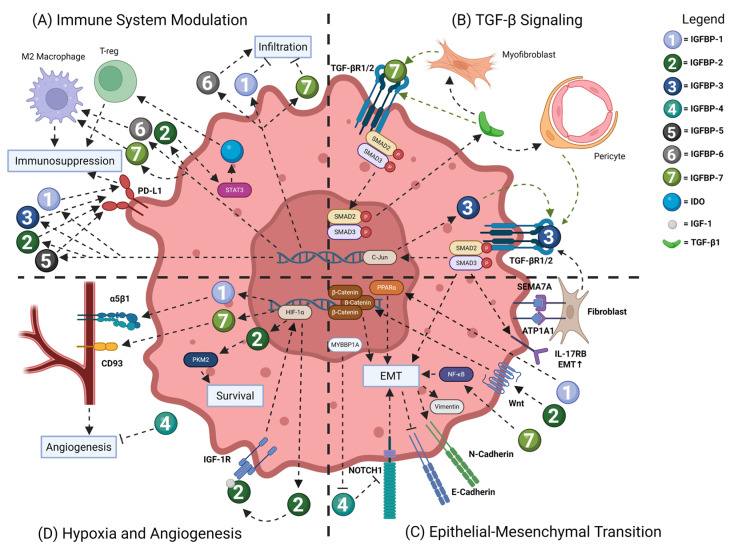
IGFBP family members play a complex role in regulating cancer cell growth, survival and migration. (**A**) Tumor-secreted IGFBP family members regulate the infiltration of pro-inflammatory immune cells and induce phenotype switching to a more immunosuppressive environment, promoting tumor growth. Moreover, IGFBP family members can elevate PD-L1 expression, exacerbating immunosuppressive phenotypes. (**B**) IGFBP-3 and 7 display a complex feed-forward relationship with TGF-β wherein TGF-β1 induces myofibroblast expression of IGFBP-7 and pericyte expression of IGFBP-3 that activates TGF-β receptors and downstream SMAD2/3 signaling. Note: green arrows represent direct pro-tumor growth signaling. (**C**) IGFBP family members regulate tumor cell migration and epithelial-to-mesenchymal transition (EMT) through a variety of signaling cascades. EMT potential is measured in the increase in mesenchymal markers N-Cadherin and Vimentin and the reduction in the epithelial marker E-Cadherin. (**D**) Hypoxic environments activate HIF-1α expression leading to IGFBP expression through direct transcription. IGFBPs promote tumor survival in a hypoxic environment. Lastly, IGFBPs in part regulate angiogenesis in tumors, further promoting tumor survival and growth. Note: dashed lines represent signaling interactions. Arrows mean positive regulation and blunt arrows mean inhibition. STAT3 = Signal Transducer and Activator of Transcription 3; PD-L1 = Programmed Death-Ligand 1; IDO = indoleamine 2; 3-dioxygenase; TGF-βR1/2 = Transforming Growth Factor Receptor 1/2; TGF-β1 = Transforming Growth Factor 1; SMAD2/3 = Mothers Against Decapentaplegic Homolog 2/3; SEMA7A = Semaphorin 7A; Peroxisome Proliferator-Activated Receptor alpha; MYBBP1A = MYB Binding Protein 1a; NOTCH1 = Neurogenic Locus Notch Homolog Protein 1; IGF-1R = Insulin-Like Growth Factor 1 Receptor; HIF-1α = Hypoxia-Inducible Factor 1-alpha; PKM2 = Pyruvate Kinase M2.

Cancer, in the most basic terms, is the uncontrolled growth of a cell through a dysregulation of homeostatic signaling pathways. As such, cancer is a multifactorial disease defined by reproducible and consistently dysfunctional mechanisms coined the hallmarks of cancer [27]. Understanding the key signaling factors responsible for the propagation of the hallmarks can lead to the development of novel therapeutics to reduce tumor burden in patients and potentially ameliorate crippling comorbidities. Interestingly, the IGFBP family members have been intimately connected to cancer progression and severity [28] and many of the family members display conserved mechanisms across cancer types. Herein, we describe how IGFBPs can regulate the Immune system, TGF-β growth signaling, hypoxia, angiogenesis, and epithelial-to-mesenchymal transition (EMT) (Figure 1), all of which have been described as hallmarks of cancer and are instrumental in tumor development and progression.

### 3.1. Immune System Modulation

There is a well-established relationship between immune cell dysfunction and tumor progression in various forms of cancer. Aberrant inflammation promotes tumorigenesis [29], and the tumor microenvironment secretes various cytokines that induce an anti-inflammatory environment, further promoting survival [30]. Understanding the signaling mechanisms that promote anti-inflammatory tumor-associated macrophages (TAMs), neutrophils, and regulatory T cells is crucial for developing successful cancer therapies [31]. Importantly, there is a defined relationship between increased immune cell infiltration into the tumor microenvironment and improved patient outcomes. A comprehensive TCGA screening study found a complex relationship between overall survival, immune cell infiltration and IGFBP-1 levels. Although context- and cancer-dependent, elevated IGFBP-1 correlated with reduced cell infiltration and therefore worsened patient outcomes in ovarian, lung, stomach and pancreatic cancer [32]. In contrast, low IGFBP-1 expression correlates with improved survival in stomach cancer and inversely correlates with CD4+ and CD8+ T cells, dendritic cells and neutrophils [33]. In renal cell carcinoma, elevated IGFBP-1 correlates with worsened survival and is inversely proportional to CD14 staining in tumor samples, indicating reduced macrophage and monocyte infiltration [34]. Interestingly, in breast cancer, where elevated IGFBP-6 correlates with improved survival, TCGA analysis found that IGFBP-6 expression also positively correlated with CD4+ and CD8+ T-cell and macrophage infiltration into the tumor microenvironment (TME) [35]. Lastly, although IGFBP-7 overexpression is associated with worsened patient survival in glioma [36], bladder [37], and stomach cancer [38], it induces both pro- and anti-inflammatory immune cell infiltration. More work is needed to define a role for IGFBP-7 in regulating phenotype switching of immune cells in cancer.

Another key factor in cancer progression and outcomes is the phenotypic state of immune cells within the TME. Two independent studies demonstrate that IGFBP-2 correlates with worsened survival in pancreatic ductal adenocarcinoma (PDAC) and that IGFBP-2 stimulates STAT3 signaling, leading to immune suppression phenotypes. Overexpression and knockdown studies of IGFBP-2 in PDAC cell lines show that IGFBP-2 activates STAT3 signaling in PDAC cells and elevates indoleamine 2, 3-dioxygenase (IDO) secretion, a known inducer of T-reg cells. As such, increased T-reg infiltration in the TME is associated with worsened survival [39]. Furthermore, similar experiments by this group define a role for IGFBP-2 in activating STAT3 signaling and subsequent macrophage polarization to an immunosuppressive M2 phenotype [40]. In colorectal cancer (CRC), a similar paradigm is reported. Fibroblasts from metastatic lesions in CRC secrete elevated IGFBP-2 that correlates with CD38 expression. In culture, these cells induced an immunosuppressive phenotype in macrophages and T cells, which was abrogated via siRNA gene silencing of IGFBP-2 [41]. Furthermore, the same group showed that in a murine model of CRC, CD38/IGFBP-2-expressing fibroblasts promote tumor growth and survival via lowered immune cell activation [42]. Fibroblast-secreted IGFBP-7 has also been shown to promote macrophage polarization through FGF2/AKT signaling mechanisms and reduce survival outcomes in gastric cancer [43]. Similar responses have been shown in glioma. IGFBP-2 and 6 promote M2 polarization, promoting tumor progression, and knockdown mouse models identified a phenotype switch to pro-inflammatory M1 cells and reduced tumor growth [44,45].

One interesting, common mechanism in the literature is the modulation of PD-L1 expression in cancer cells and select immune cells. PD-L1 or programmed cell death ligand 1 acts to inhibit a T-cell response, propagating tumor survival [46]. In brief, IGFBP-1 [47], IGFBP-2 [48], IGFBP-3 [49], and IGFBP-5 [50] have been shown to elevate PD-L1 expression on macrophages and tumor cells, leading to reduced immune response in gliomas, esophageal, and colorectal cancers. Furthermore, IGFBP-1 and PD-L1 co-overexpression sensitizes patients to checkpoint inhibitor therapy [47]. Collectively, there are convincing reports related to overexpression of IGFBP family members and induction of an immunosuppressive TME. Moreover, IGFBP overexpression may indicate a patient’s response to emerging immunotherapies. Collectively, IGFBPs have been shown to regulate both immune cell invasion in cancer and manipulate immune cell phenotype to induce a pro-tumor environment (Figure 1A). Moreover, there is an opportunity to combine previously established immunotherapies with anti-IGFBP therapies for the treatment of various cancer types.

### 3.2. TGF-β Signaling

There is an emerging field of research describing IGFBPs’ interactions with TGF-β signaling pathways, which are known to play a complex role in tumor progression [51]. Of note, there are two studies implicating a role for IGFBPs binding directly to TGF-β receptors. In glioblastoma, IGFBP-3 can interact directly with TGF-β receptors, stimulating SMAD2/3 signaling cascades and subsequent c-Jun activation. This promotes positive feedback on IGFBP-3 expression, and continual IGFBP-3 overexpression promotes cancer progression [52]. In vitro analysis using an esophageal carcinoma cell line indicated that increasing IGFBP-7 using an adenovirus system can directly induce SMAD2/3 activation and promote expression of TGF-β1, positively associated with tumor development [53]. Lastly, it is important to acknowledge a relationship whereby TGF-β regulates IGFBP expression. A complex mechanism has been described where CRC-derived tumor cells secrete TGF-β1 which induces IGFBP-3 expression in pericytes. This effect is negated in the presence of a TGF-β receptor antagonist. IGFBP-3 expression in pericytes positively regulates tumor cell proliferation and migration [54]. A similar crosstalk system has been found in gastric cancer. Tumor-derived TGF-β1 induces IGFBP-7 in myofibroblasts, which positively regulates tumor proliferation and metastasis and can be attenuated by TGF-β receptor antagonists [55]. Curiously, IGFBP-3 and IGFBP-7 have been found to be induced by TGF-β1 expression and activate TGF-β receptors in a tumor context. Given the complex nature of TGF-β signaling in cancer (Figure 1B), work must be conducted to further elucidate these complex interactions and identify potential therapeutic targets.

### 3.3. Epithelial-to-Mesenchymal Transition

IGFBP family members were first discovered for their roles in interacting with IGF proteins, potent regulators of various metabolic processes in cells [2]. Metabolic dysfunction is one of the original cancer hallmarks and plays a complex role in tumor progression [27,56]. As such, there is an abundance of literature identifying IGFBP members as metabolic regulators of tumor growth. Here we will focus on one metabolic mechanism, epithelial-to-mesenchymal transition (EMT), where, in fact, all but IGFBP-5 and 6 have been implicated. EMT is a process that allows epithelial cells to lose their polarity and acquire the phenotype of a mesenchymal-like cell and is a necessary step for a tumor to become metastatic. Importantly, EMT is characterized by a loss of epithelial markers like E-Cadherin and increased expression of mesenchymal markers like N-Cadherin and Vimentin among others [57]. To date, there are no cancer therapies that modify or inhibit EMT to reduce metastatic potential [58,59].

In lung adenocarcinoma cell lines where elevated IGFBP-1 correlates with reduced survival, knockout of IGFBP-1 significantly reduces cell proliferation and migratory potential in vitro. This is accompanied by reductions in pro-mesenchymal markers vimentin and N-cadherin and increases in the epithelial marker E-cadherin. It was reported that IGFBP-1 knockdown reduced PPARα expression and overexpressing PPARα pharmacologically overcame the IGFBP-1-dependent changes in proliferation and migration. However, subsequent in vivo models using IGFBP-1-null cells showed no significant reduction in metastatic nodules, indicating a compensatory mechanism in vivo [60]. Fibroblast secretion of IGFBP-2 in aged models has been found to be a potent promoter of melanoma metastasis in vivo and cell migration in vitro [61]. Two reports found IGFBP-2 to regulate EMT in hepatocellular carcinoma (HCC). One found that highly metastatic HCC tumors secrete extracellular vesicles rich in IGFBP-2. Administration of these vesicles further induced EMT in an HCC cell line with reduced metastatic potential and elevated vimentin and reduced E-cadherin. This elegantly identified IGFBP-2 as a primary inducer of EMT in HCC [62]. Moreover, overexpression of IGFBP-2 in mouse models of HCC increased markers of EMT vimentin and N-cadherin and was dependent on Wnt/β-catenin signaling and nuclear accumulation of β-catenin [63]. Interestingly, studies in melanoma demonstrate that in early phases of tumor growth and metastasis where IGFBP-3 expression is reduced due to promoter hypermethylation [64], elevated IGFBP-3 inhibits Wnt/β-catenin signaling, reducing metastatic potential [65]. However, during late-stage growth in highly metastatic melanoma cell lines, IGFBP-3-promoted cell migration and knockdown reduced cell motility [66]. Furthermore, an incredible study in PDAC using high-resolution immunofluorescence demonstrated that through SEMA7A and ATP1A1 interactions, tumor cells promote fibroblast secretion of IGFBP-3. The fibroblast-secreted IGFBP-3 subsequently acts on tumor cells to upregulate IL-17RB at the metastatic front in the tumor and promote invasion. Furthermore, this group confirmed IGFBP-3 interactions with TGF-β receptor 2 and determined that interaction is essential for IL-17RB secretion and invasion [67]. IGFBP-3 has also been implicated in promoting EMT and migration in tongue squamous cell carcinoma. Using cell tracking software and SAS-Fucci cells, IGFBP-3 knockdown has been shown to reduce cell motility and induce cell cycle arrest, slowing proliferation [12]. Likewise, IGFBP-7 has a biphasic response in gastric cancer proliferation and metastatic potential. Overexpression of IGFBP-7 accelerates tumor proliferation and invasion in vitro, whereas a knockdown reduces proliferation and metastatic potential. A similar trend was shown in in vivo models of gastric cancer [68]. On the contrary, much like in potentiating angiogenic potential described below, IGFBP-4 has a negative impact on EMT in HCC. IGFBP-4 overexpression inhibits EMT, induces E-cadherin, and reduces vimentin and N-cadherin expression. The opposite was true for models of IGFBP-4 silencing. In summation, IGFBPs play a crucial role in regulating tumor EMT and metastasis, a currently untreated consequence of tumor progression (Figure 1C). Further understanding the highlighted mechanisms of action may identify novel therapeutic targets for metabolic regulation and controlling metastasis.

### 3.4. Hypoxia and Angiogenesis

Hypoxia is a key feature of most solid tumors. Cancer cells readily adapt to the lack of oxygen in a growing tumor, enabling continued growth and metastasis. The best-defined transcription factors regulating cancer cells’ response to hypoxia are HIF-1α and HIF-1β [69]. In a model of endometrial cancer where IGFBP-2 overexpression correlates with disease progression, hypoxic environments significantly mediate IGFBP-2 overexpression and allow HIF-1α to bind to its promoter. HIF-1-induced overexpression of IGFBP-2 led to elevated PKM2 expression and a significant shift towards glycolysis, enabling tumor survival in hypoxic environments [70]. An independent study with anaplastic Wilms tumor demonstrated that HIF-1α interacts with the IGFBP-2 promoter and HIF-1 knockout significantly reduces IGFBP-2 expression. Moreover, a positive feedback loop exists where IGFBP-2 induces expression through IGF-1-mediated signaling [71]. Interestingly, tumor-expressed HIF-1 regulates skeletal muscle wasting via promotion of IGFBP-5 expression. In an elegant study using two model organisms for cancer cachexia, it was shown that *ImpL2* in *Drosophila,* a direct homolog to IGFBP-5 in mammals, is under the regulation of HIF-1α and directly promotes muscle loss. Genetic deletion of HIF-1 in tumors in fly and mouse models significantly reduced IGFBP-5 expression and the loss phenotype [72]. Together, these studies demonstrate a need to interrogate HIF-1:IGFBP signaling mechanisms to identify combination therapies in the treatment of various cancers and potential comorbidities.

Hypoxia is intricately connected to angiogenesis which plays a complex role in tumor progression. Although largely context-dependent, inhibiting angiogenesis in tumors aids in slowing growth [69,73]. Recently, a few examples of IGFBP family members modulating angiogenesis have been presented. In hepatocellular carcinoma tumors treated with anti-angiogenic therapy, hypoxia increases IGFBP-1 and promotes angiogenesis and tumor growth. Dual treatment targeting angiogenesis and IGFBP-1 significantly reduced tumor burden, and the mechanism was attributed to IGFBP-1/α5β1 integrin interactions in endothelial cells [74]. In models of melanoma, it was found that HIF-1α activation downstream of melanoma differentiation-associated gene 9 induced expression of IGFBP-2, which promoted endothelial cell migration into the tumor, increasing VEGF-A expression and angiogenesis, enhancing tumor survival [75]. Moreover, novel interactions between tumor-secreted IGFBP-7 and CD93 on endothelial cells led to reduced angiogenesis and slowed tumor growth in in vitro models of melanoma, confirming the role of IGFBP-7:CD93 interactions in tumor development [76]. On the contrary, IGFBP-4 was identified as a potent inhibitor of angiogenesis in models of squamous cell carcinoma where IGFBP-4 neutralization improved angiogenesis and tumor outgrowth [77]. Collectively, a unique connection between hypoxia, IGFBP expression, and angiogenic potential has been established and defines a potential for targeted combination of IGFBP and hypoxia therapies in cancer (Figure 1D).

## 4. IGFBP Signaling Pathways in Vascular Homeostasis and Pathology

### 4.1. Hypoxia and Angiogenesis

**Figure 2:**
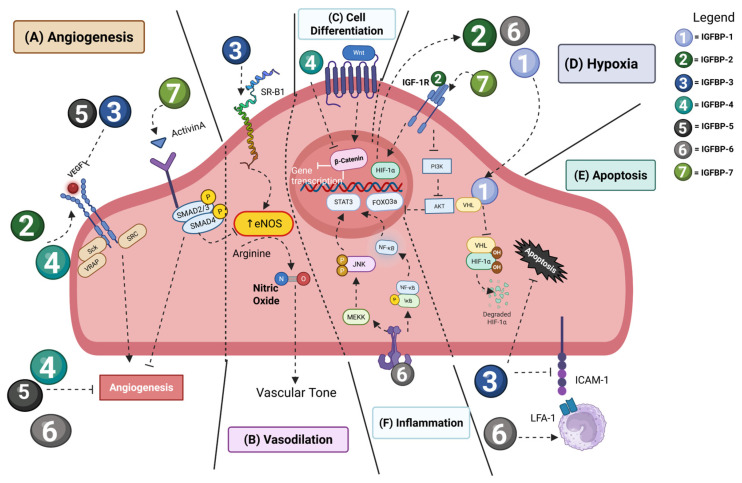
IGFBPs’ role in vascular homeostasis and cardiovascular disease. (**A**) Angiogenesis—IGFBP-2 is pro-angiogenic and promotes VEGF transcription. IGFBP-4, 5, and 6 inhibit angiogenesis; however, IGFBP-4 promotes VEGF-mediated angiogenesis in myocardial infarction. IGFBP-3 and 5 inhibit VEGF signaling. IGFBP-7 binds to Activin A and inhibits angiogenesis. (**B**) Vasodilatation—IGFBP-3 contributes to the secretion of nitric oxide by binding to the scavenger receptor class B, type 1, thereby regulating vascular tone. (**C**) Cell differentiation—IGFBP-4 enhances cell differentiation by inhibiting β-catenin signaling. (**D**) Hypoxia—IGFBP-2 binds to IGF-1R and promotes HIF-1α transcription. HIF-1α regulates the expression of IGFBP-1, 2 and 6. IGBP-7 inhibits FOXO3a expression via the PI3K/AKT pathway, inducing senescence. (**E**) Apoptosis—IGFBP-1 is upregulated by HIF-1α, which in turn contributes to their apoptosis. IGFBP-3 is involved in ICAM1 suppression, preventing monocyte adhesion and limiting endothelial apoptosis. IGFBP-6 increases monocyte adhesion and apoptosis. (**F**) Inflammation—IGFBP-6 is pro-inflammatory. Via the JNK and NF-kB signaling, it is known to increase inflammation in the endothelial cells. Note: dashed lines represent signaling interactions. Arrows mean positive regulation and blunt arrows mean inhibition. STAT3 = Signal Transducer and Activator of Transcription 3; FOXO3a = Forkhead box O3; SMAD2/3 = Mothers Against Decapentaplegic Homolog 2/3; IGF-1R = Insulin-Like Growth Factor 1 Receptor; HIF-1α = Hypoxia-Inducible Factor 1-alpha; JNK = c-Jun NH-2 terminal kinase; MEKK = mitogen-activated protein kinase (MEK) kinase; VEGF = Vascular Endothelial Growth Factor; Sck = SH3 Domain-Containing Kinase-Binding Protein 1; VRAP = VEGF Receptor-Associated Protein; SRC = Sarcoma protein kinase; SR-B1 = scavenger receptor class B, type 1.

The IGF signaling pathway plays an essential role in cardiac development, with IGF2 representing a primary inducer of cardiomyocyte proliferation and morphogenesis of the myocardial wall [78]. Beyond this, IGFBPs can modulate pathologic cardiac development (Figure 2). For example, IGFBP-3 secreted by coronary endothelial cells contributes to decreased cardiomyocyte proliferation and increased maturation and the development of abnormal myocardial compaction. IGFBP-4 enhances cardiomyocyte differentiation from stem cells and promotes proliferation by inhibition of β-catenin signaling [79] (Figure 2C). Endothelial cells (ECs) express IGFBP-2, IGFBP-3, IGFBP-4, IGFBP-5 and IGFBP-6, with IGFBP-2 and IGFBP-3 predominating in microvascular ECs, and IGFBP-3 and IGFBP-4 playing larger roles in ECs from large vessels. IGFBP-2 is pro-angiogenic, IGFBP-4, IGFBP-5 and IGFBP-6 inhibit angiogenesis, and there is evidence for both pro-angiogenic and anti-angiogenic action by IGFBP-3 and IGFBP-7 [80] (Figure 2A). IGFBP-2 increases angiogenesis via transcriptional regulation of VEGF, whereas IGFBP-3 and IGFBP-5 inhibit VEGF signaling, and IGFBP-6 is induced by hypoxia and may serve as a negative regulator of HIF-1α signaling. IGFBP-3 and IGFBP-4 are important in sprouting angiogenesis, with IGFBP-3 loss of function leading to decreased angiogenesis, whereas knockdown of IGFBP-4 results in increased angiogenesis [81].

### 4.2. Cardiovascular Disease

Hypoxia upregulates IGFBP-1 in cardiomyocytes, which mediates their apoptosis, and this is regulated by HIF-1α [82]. Moreover, IGFBP-1 promotes neovascularization in response to ischemia with deletion of IGFBP-1 inhibiting endothelial regeneration following injury, while developmental angiogenesis is not affected by IGFBP-1 [83]. IGFBP-1 is also associated with increased risk of major cardiovascular events in patients with peripheral arterial disease [84] (Figure 2D).

In pulmonary hypertension, in addition to their IGF-dependent signaling, IGFBP-1 and IGFBP-2 have an IGF-1R-independent kinase activation pattern in smooth muscle cells, and IGFBP-2 induces EGFR and STAT3 signaling [85]. IGFBP-2 is also a potential biomarker for heart failure development and severity [86].

IGFBP-3 is downregulated in aortic tissues from patients with aortic dissection, and this is mediated by IGFBP-3 preserving aortic smooth muscle cells’ contractility and by reducing MMP9 activation [87]. In retinopathy of prematurity, IGFBP-3 increased endothelial migration, VEGFR1 and VEGFR2 expression, tube formation, and NO synthesis (Figure 2B) [88,89]. In diabetic retinopathy, IGFBP-3 reduced endothelial apoptosis and endothelial-mediated monocyte adhesion in an IGF-independent manner by suppressing ICAM1 [90] (Figure 2E). IGFBP-3 levels are increased in rats undergoing cardiac ischemia and represent a potential biomarker for coronary artery disease [91].

IGFBP-4 enhances cardiomyocyte differentiation while its absence impairs differentiation, and this is mediated through Wnt signaling [92]. Moreover, it can induce VEGF-mediated angiogenesis during myocardial infarction [93]. IGFBP-4’s actions in cardiovascular disease are also mediated by its cleavage by PAPP-A and MMPs, which causes accumulation of IGF on the outer membrane of cardiomyocytes [94].

Cardiomyocyte-specific IGFBP-5 knockdown inhibited myocardial apoptosis and increased cardiomyocyte proliferation in mice with myocardial infarction. IGFBP-5 also ameliorated pathological cardiac remodeling, and this was mediated by IGF-1-dependent signaling [95]. Functionally, IGFBP-5 deficiency protects against ischemic injury by promoting ATP metabolism and stabilizing HIF-1α [96].

IGFBP-6 plays an important role in regulating smooth muscle cell proliferation; knockdown of IGFBP-6 attenuated cell proliferation and increased cyclin E ubiquitination and phosphorylation of CDK2 [97]. Moreover, IGFBP-6 levels are reduced in atherosclerotic arteries, and a reduction in IGFBP-6 in endothelial cells increases inflammatory molecule expression and monocyte adhesion but has anti-inflammatory effects mediated via JNK and NF kappa B (NF-κB) signaling [98] (Figure 2F).

IGFBP-7 is upregulated in the angiogenic vasculature during physiological vessel formation via VEGF-A [99] and pathological processes such as tumor neovascularization [100]. Importantly, IGFBP-7 also plays a critical role in heart failure. In addition to being a sensitive and specific biomarker for heart failure, IGFBP-7 mediates cardiac senescence by stimulating IGF-1R-dependent suppression of FOXO3a [101,102] (Figure 2D). Excitingly, endothelial-specific IGFBP-7 knockout and therapeutic targeting of IGFBP-7 using a vaccine-based approach both ameliorated cardiac dysfunction in a pressure overload model of heart failure [103]. In acute lung injury, IGFBP-7 is overexpressed in endothelial cells, and this allows increased infiltration of Clec4n^hi^ neutrophils to the lung which mediate barrier dysfunction [104].

## 5. IGFBP Signaling Pathways in Fibrosis

**Figure 3:**
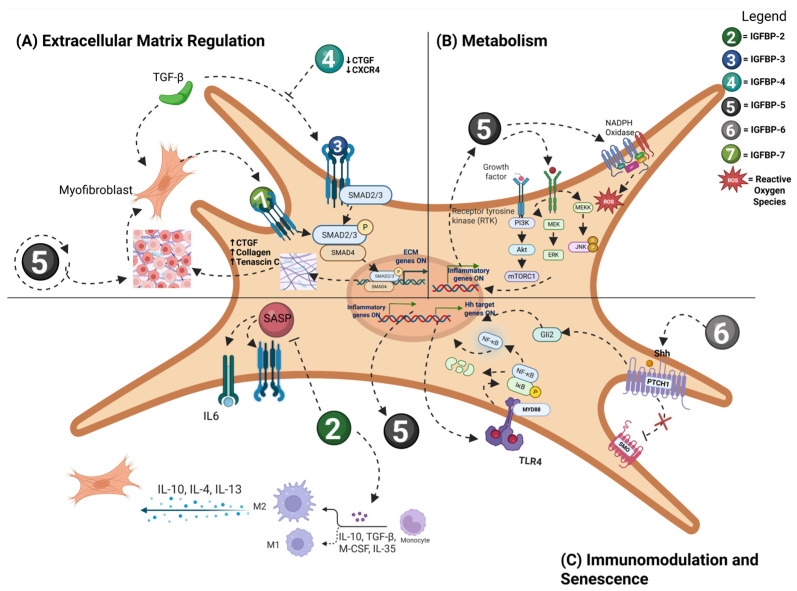
IGFBPs’ involvement in fibrotic mechanisms in fibroblasts and mesenchymal cells. (**A**) Extracellular matrix regulation—IGFBP-3 and IGFBP-7 activate TGF-β receptors and downstream SMAD signaling, causing an increase in expression of Collagen, CTGF, and Tenascin C, enabling ECM deposition in fibrosis. TGF-β-induced myofibroblasts are involved in a self-amplifying loop of activating IGFBP-7 secretion and TGF-β receptors. IGFBP-5 promotes myofibroblast differentiation and fibrosis. It increases pro-fibrotic genes and ECM deposition. IGFBP-5’s ability to self-amplify its own expression adds to the fibrotic tissue microenvironment. IGFBP-4, however, blocks TGF-β-induced ECM production and deposition. It also reduces CTGF and CXCR4 levels. (**B**) Metabolism—IGF signaling via IGFBPs binding to IGFRs leads to downstream PI3K/AKT/mTOR, MEK/ERK, and JNK signaling. IGFBP-5 enables the MEK/ERK and JNK signaling that induces ROS production. This is also mediated by NADPH oxidase. (**C**) Immunomodulation and senescence—IGFBP-2 induces M2 polarization of macrophages, which contributes to a pro-inflammatory environment. Inflammatory cytokines from M2 macrophages stimulate myofibroblasts, which then create a pro-fibrotic condition. IGFBP-2 also inhibits SASP, including pro-inflammatory cytokines, chemokines, growth factors, proteases, and ECM components. IGFBP-6 induces myofibroblast differentiation by activating the sonic hedgehog pathway that, in turn, induces TLR4 signaling, whose downstream effects include the NF-kB pathway and activating inflammatory genes. Note: dashed lines represent signaling interactions. Arrows mean positive regulation and blunt arrows mean inhibition. ECM = extracellular matrix; CTGF = connective tissue growth factor; SASP = senescence-associated secretory phenotype; SMAD2/3 = Mothers Against Decapentaplegic Homolog 2/3; TLR4 = Toll-like receptor 4; IL = interleukins; TGF-β = Transforming Growth Factor 1; JNK = c-Jun NH-2 terminal kinase; MEKK = mitogen-activated protein kinase (MEK) kinase; ROS = reactive oxygen species.

Fibrosis is a pathologic development of scar tissue which involves activation of fibroblasts, increased production and deposition of extracellular matrix (ECM) components, and differentiation of fibroblasts into myofibroblasts. Fibrotic responses are mediated by a variety of growth factors and cytokines, classically including transforming growth factor-β (TGF-β). There is an increasing recognition that several insulin-like growth factor-binding proteins are associated with fibrotic conditions, and emerging themes include IGFBP regulation of TGF biology, as well as a number of emerging pathways (Figure 3). Moreover, these molecules are secreted in the circulation and represent promising biomarkers for fibrosis. IGFBPs are implicated in the fibrotic response across organs and cell types (albeit with cell-specific and organ-specific manifestations), making the IGFBP system ripe for development of novel anti-fibrotic approaches.

IGFBP-1 does not have strong mechanistic data supporting its role in fibrogenesis. There has been evidence that it contributes to non-alcoholic fatty liver disease, a precursor to hepatic fibrosis, by reducing free-fatty-acid-induced lipid accumulation via interacting with integrins and ameliorating inflammation by inhibiting NF-κB and ERK signaling pathways, and has been shown to be a risk factor for more severe fibrosis in this disease [105,106]. Some studies have observed elevated serum IGFBP-1 levels in idiopathic pulmonary fibrosis (IPF), along with a decrease in IGFBP-1 following anti-fibrotic treatment [107]. However, other studies suggest that despite elevated IGFBP-1 levels, anti-fibrotic treatment does not cause a significant impact on IGFBP-1 expression in idiopathic pulmonary fibrosis (IPF) [108].

IGFBP-2 transgenic mice have reduced senescence in alveolar epithelial type 2 (AEC2) cells and have reduced bleomycin-induced lung fibrosis; moreover, recombinant IGFBP-2 is anti-fibrotic in bleomycin lung fibrosis, and IGFBP-2 expression is decreased in AEC2 cells isolated from fibrotic lung regions of patients with IPF. Interestingly, one mechanism by which IGFBP-2 may act is on myeloid cells; in cancer-associated fibroblasts, IGFBP-2 promotes glioma progression through induction of M2 macrophage polarization (Figure 3C) [44]. Clinical studies show that serum IGFBP-2 levels are elevated in patients with IPF [108]. Additionally, elevated serum IGFBP-2 levels have been detected in systemic sclerosis-associated interstitial lung disease (SSc-ILD), and are associated with deterioration of lung function, while treatment with anti-fibrotic agents pirfenidone and nintedanib reduces serum IGFBP-2 [109]. IGFBP-2 appears to be a strong candidate biomarker for early detection and disease monitoring in IPF and SSC-ILD. Notably, IGFBP-2 levels can be measured via ELISA using sputum samples, offering a less invasive method for diagnosis and disease monitoring [110].

IGFBP-3 is known to regulate TGF-β in cancer cells but has not been widely reported in fibrotic disease. IGFBP-3 is epigenetically regulated by METTL3 to promote cardiac fibroblast activation and fibrosis in a variety of cardiac fibrosis models [111]. IGFBP-3 treatment has been shown to stimulate human primary lung fibroblasts to produce tenascin-C and Collagen 1, supporting a role for IGFBP-3 in promoting ECM deposition in pulmonary fibrosis (Figure 3A) [112]. Increased levels of IGFBP-3 have been observed in primary skin fibroblast cultures from systemic sclerosis (SSc) patients [113]. Another study detected elevated IGFBP-3 in the bronchoalveolar lavage fluid of IPF patients [114].

IGFBP-4 blocks TGF-β-induced ECM production and inhibits ECM production in lung and skin cells in vitro, while IGFBP-4 knockout reduces bleomycin-induced fibrosis. IGFBP-4 also reduces the chemokine receptor CXCR4 and the pro-fibrotic factor CTGF [115]. IGFBP-4 has not been reported to be a biomarker in fibrotic disease.

IGFBP-5 knockdown blocks myofibroblast differentiation and decreases cardiac fibrosis in animal models through interactions with NFAT4, which stimulates the production of MFAP5 and enables SREBP2-dependent cholesterol synthesis [116]. In lung fibroblasts, IGFBP-5 promotes fibrosis by increasing the production of extracellular matrix (ECM) genes and the expression of pro-fibrotic genes including connective tissue growth factor (CTGF) and lysyl oxidase [117]. Reactive oxygen species (ROS) production was induced by IGFBP-5, regulated by MEK/ERK and JNK signaling, and mediated by NADPH oxidase. Notably, IGFBP-5 can amplify its own expression, functioning as a self-reinforcing driver of fibrosis [117]. Silencing IGFBP-5 in scleroderma and IPF fibroblasts reduced production of reactive oxygen species [118]. Upregulation of IGFBP-5 is detected in the skin of SSc patients and lung tissues of IPF patients (Figure 3B) [117].

IGFBP-6 expression is increased in myelofibrosis, and IGFBP-6-induced myofibroblast differentiation is associated with an upregulation of cancer-associated fibroblast markers. This process was mediated by TLR4 signaling and the sonic hedgehog (SHH) pathway [119]. IGFBP-6 has also been implicated in a wide variety of fibrotic diseases of the skin, heart, lung, kidney, and liver, and contributes to cancer stroma in the tumor microenvironment which is mediated at least partially by interactions with TGF- β [120,121]. In Dupuytren’s disease, a hereditary form of tendon fibrosis, IGFBP-6 is downregulated [122]. In hepatic fibrosis, IGFBP-6 has emerged as a key marker; reduced IGFBP-6 expression is seen in chronic hepatitis C as it progresses to fibrosis, and in NAFLD patients with fibrosis, elevated hepatic IGFBP-6 levels strongly correlate with advanced fibrosis [123,124]. Notably, IGFBP-6 levels are also sensitive to NAFLD treatments like tesamorelin, which prevents fibrosis progression, thus confirming its clinical relevance as a biomarker [120].

Urinary IGFBP-7 (along with TIMP-2) has been reported as a well-validated biomarker for renal fibrosis [125]. IGFBP-7 knockdown attenuates renal fibrosis and improves renal function through activation of ERK1/2 signaling [126]. Renal tubular epithelial cell IGFBP-7 interacts with PKM2 to drive renal lipid accumulation which promotes fibrosis [127]. In a zebrafish model of metabolic-associated liver fibrosis, IGFBP-7 promoted fibrosis by inhibiting hepatic ferroptosis [128]. Cardiac fibroblasts regulate cardiomyocyte homeostasis, but under pressure overload, they mediate cardiac fibrosis through the Htra3-TGF-β-IGFBP-7 pathway [129]. Importantly, there is a positive feedback loop between IGFBP-7 and TGF-β1: TGF-β inhibition significantly reduced IGFBP-7 expression, while IGFBP-7 knockout suppressed TGF-β1 expression [130]. IGFBP-7 is increased in the skin of patients with systemic sclerosis, and correlates with the modified Rodnan skin score, a validated measure of skin fibrosis severity [131]. IGFBP-7 is also a potential marker for early diagnosis of IPF in which elevated IGFBP-7 expression is seen in lung epithelial cells [130].

## 6. IGFBPs as Biomarkers of Disease

Given preclinical data suggesting that IGFBPs can regulate the progression of cancer, vascular disease, and fibrosis through both IGF-1-dependent and -independent mechanisms, their role as biomarkers in a clinical setting has been investigated. First, an understanding of which tissues express IGFBPs at homeostasis and during various pathological states, identified via The Human Protein Atlas, can aid in early detection and guide the development of targeted therapeutics to improve patient outcomes and quality of life. IGFBP-1 shows high RNA expression in the liver and lower levels in female reproductive tissues such as the ovary and endometrium, with protein expression predominantly localized to the placenta. Its moderate hepatic and endometrial expression patterns are consistent with reports linking IGFBP-1 to lung, endometrial, and breast cancers [132]. IGFBP-2 demonstrates moderate RNA expression in the brain, prostate, and liver, but exhibits higher levels in the pancreas and stomach. IGFBP-2 is frequently upregulated in gliomas, prostate cancer, and hepatocellular carcinoma, consistent with its basal expression in these organs [133,134]. IGFBP-3 shows the highest RNA expression in the placenta and liver, with basal expression in most other tissues except the cerebellum and bone marrow. Its broad expression in the breast, lung, colorectum, and prostate correlates with cancers of these organs [135,136]. IGFBP-4 displays high RNA levels in the liver and endometrium and moderate expression in the heart, lung, ovary, and uterus, strengthening its association with lung, ovarian, and uterine malignancy [137]. IGFBP-5 exhibits strong RNA expression in female reproductive tissues, including the cervix, ovary, and fallopian tube, with variable levels in other organs. This aligns with its reported roles in breast and lung cancers as well as in fibrotic conditions [137,138]. IGFBP-6 presents high protein expression in the brain, thyroid gland, lymphoid organs, and gastrointestinal tract, with RNA levels peaking in the choroid plexus and reproductive tissues. These patterns correspond with its reported associations with ovarian and brain tumors [139]. IGFBP-7 demonstrates moderate RNA expression in the prostate, lung, skin, and vascular tissues, and higher levels are observed in the choroid plexus, kidney, heart, and thyroid gland. Its tissue expression correlates with its reported roles in melanoma, prostate, and vascular cancers [140,141]. As such, Table 1 summarizes findings from 2020 to now on the clinical expression patterns, prognostic significance, and impact on survival outcomes of IGFBP family members in common cancer diagnoses: breast, lung, pancreatic, and gastrointestinal, and various vascular and fibrotic diseases.

## 7. Summary

The IGFBP family of proteins is traditionally known for their roles in regulating canonical IGF and insulin signaling in a variety of tissue types, helping to maintain metabolic homeostasis. Canonically, IGFBPs function to bind the mitogens IGF1/2 to regulate their binding to IGFRs which control biological mechanisms such as cellular growth, differentiation, and apoptosis. More recently, these proteins have gained attention for a variety of non-canonical signaling types in pathological pathways independent of IGF activity that have more disease potential. Their non-canonical binding of integrins and nuclear translocation results in several pathologies such as cellular senescence, apoptosis, inhibition of cell differentiation and gene transcription, tumorigenesis and tumor cell proliferation, angiogenesis, cardiovascular disease, and fibrosis. In addition to the canonical and non-canonical functions of IGFBP family members, research suggests that due to their role in the onset, development, and/or progression of certain diseases, they may act as effective biomarkers of disease state, severity, and prognosis. The dysregulation in IGFBP secretion is multifactorial and disease-specific, and a more nuanced understanding of this system is needed to understand how the similarities and differences in IGFBP family members relate to these unique and shared pathologies. However, the pathways that lead to disease are largely conserved across pathologies. Dysregulation in immune system function, TGF-β signaling, hypoxia and angiogenesis, and inflammation are commonly associated with pathological secretion of IGFBP family members. More preclinical and clinical research is needed to clearly define the role of IGFBPs in cancer diagnosis, cardiovascular disease, and fibrosis to determine their viability as therapeutic targets, especially because they do play vital roles in homeostasis. While anti-IGFBP antibodies have shown promise preclinically, there have yet to be clinical trials with these kinds of targeted therapies. Additionally, more information is needed to identify ways in which these complicated systems regulate disease. Specifically, as it pertains to cancer, research should focus on isolating the cells in the tumor microenvironment responsible for the secretion of IGFBPs and in which cancers targeting IGFBPs might have therapeutic potential to ameliorate disease, reduce disease-related toxicities, improve quality of life, and increase survival. Although much research has been performed on the physiological production and secretion of IGFBPs, more research is needed to elucidate the conditions in which the physiological production becomes pathological and leads to subsequent conditions of organ dysfunction such as interstitial lung disease, atherosclerosis, scleroderma, skeletal muscle wasting, and sarcopenia. Mechanistically, the identification of pathways that are dysregulated in the environment of increased or decreased IGFBP production may allow for the development of therapeutics that can target multiple proteins downstream of their secretion.

## Figures and Tables

**Table 1:** IGFBP disease outcomes in cancer, cardiovascular disease and fibrosis.

	Disease	Tissue Type	Expression	Sample Size	Response to Treatment/Longitudinal Study Results	References
IGFBP-1	Colorectal Cancer	Serum and tumor tissue	Increase	328	Poor survival	[142]
	Esophageal Cancer	Serum	Increase	1064	-	[143]
	Gastric Cancer	Tumor tissue	Increase	11,084	Poor survival	[144]
	Lung Cancer	Tumor Tissue	Increase	9736	Poor survival	[145]
	Idiopathic pulmonary fibrosis (IPF)	Serum	Increase	72, 50	Levels increased with time/severity, insensitive to anti-fibrotic treatments	[107,108]
	Non-alcoholic fatty liver disease (NAFLD)	Serum	Increase	52	More advanced fibrosis	[106]
	Heart failure with reduced ejection fraction	Serum	Increase	250	Presence of heart failure and likelihood of adverse event	[146]
	Peripheral arterial disease	Serum	Increase	465	Increased risk of major adverse cardiovascular events (MACE)	[84]
IGFBP-2	Colorectal Cancer	Tumor tissue	Increase	5560	Poor survival	[147]
	Esophageal Cancer	Tumor tissue	Increase	Not reported	-	[19]
	Gastric Cancer	Serum and tumor tissue	Increase	118	Correlates with tumor stage and poor survival	[19]
	Lung Cancer	Serum and tumor tissue	Study-dependent	30, 20, 201	Poor survival in lung adenocarcinoma and better survival in lung squamous cell carcinoma	[145,147,148,149,150]
	Breast Cancer	Serum and tumor tissue	Increase	412	Poor survival and tamoxifen resistance	[19]
	Pancreatic Cancer	Serum	Increase	165	Correlates with tumor stage and poor survival	[151]
	IPF	Serum	Increase	50, 15	Elevated in patients and decreased with anti-fibrotic treatment	[108,110]
	Systemic Sclerosis (SSc)	Serum	Increase	102	Negatively correlates with pulmonary function and disease progression	[109]
	Acute coronary syndrome	Serum	Increase	277	Increased risk of MACE	[152]
	Heart failure	Serum	Increase	870	Increased risk of cardiovascular mortality	[86]
IGFBP-3	Colorectal Cancer	Tumor tissue	Increase	202	Poor survival	[153]
	Gastric Cancer	Serum and tumor tissue	Study-dependent	1541, 11,084	-	[144,154]
	Lung Cancer	Serum and tumor tissue	Study-dependent	131	Inverse relationship to tumor stage in non-small cell lung cancer, and disease risk in lung adenocarcinoma	[145,150,155]
	Breast Cancer	Serum	Increase	334,236	Increased risk of developing disease	[156,157]
	Pancreatic Cancer	Serum and tumor tissue	Study-dependent	88, 478	Serum negatively correlates and tumor expression positively correlates with survival	[158,159]
	SSc	Serum	Increase	92	-	[160]
	SSc-ILD	Lung tissue	Increase	Not reported	-	
IGFBP-4	Gastric Cancer	Tumor tissue	Increase	11,084	-	[144]
	Lung Cancer	Serum	Increase	83	-	[161]
	Breast Cancer	Tumor tissue	Decrease	162	Improved survival	[28]
	Pulmonary Arterial Hypertension (PAH)	Serum	Increase	2579	Shorter 6 min walk distance, worse NYHA functional classification, and decreased survival.	[162]
	Ischemic heart disease	Serum	Increase	1417	Increased risk of MACE and mortality	[163]
IGFBP-5	Lung Cancer	Serum	Decrease	100	Improved survival	[164]
	Colorectal Cancer	Tumor tissue	Increase	56	Correlates with tumor stage and poor survival	[165]
IGFBP-6	Breast Cancer	Tumor tissue	Decrease	1091	Improved survival	[35]
	Colorectal Cancer	Tumor tissue	Decrease	130	Improved survival	[166]
	Lung Cancer	Serum	Decrease	31	-	[167]
	Fibrosis in NAFLD	Serum and liver tissue	Increase	61	Reduced IGFBP-6 expression on anti-fibrotic tesamorelin	[124]
	Atherosclerosis	Serum and artery tissue	Decrease	3	-	[98]
IGFBP-7	Gastric Cancer	Tumor tissue	Increase	16	Poor survival	[38]
	Breast Cancer	Tumor tissue	Increase	878	Increased disease risk and poor survival	[168]
	Lung Cancer	Serum	Increase	90	Correlates with tumor stage and metastasis	[169]
	Heart failure	Serum	Increase	2250, 313	Increased risk of all three main hospitalization and mortality causes	[101,102]
	Heart failure risk	Serum	Increase	13,900	Risk of future CHF development in healthy individuals	[170]
	Pulmonary hypertension	Serum	Increase	2582	Decreased six-minute walk distance, higher mean right atrial pressure, decreased survival	[162]
	SSc	Serum	Increase	37	Higher modified Rodnan skin score (MRSS)	[131]

## Data Availability

No new data were created or analyzed in this study. Data sharing is not applicable to this article.

## Abbreviations

The following abbreviations are used in this manuscript:

AEC2	Alveolar Epithelial Type 2
ALS	Acid Labile Subunit
ARTA	All-Trans Retinoic Acid
ATP1A1	ATPase Na^+^/K^+^-Transporting Subunit Alpha 1
CAF	Cancer-Associated Fibroblast
CDK2	Cyclin-Dependent Kinase 2
CHF	Congestive Heart Failure
Clec4nhi	C-Type Lectin Domain Family 4
CRC	Colorectal Cancer
CTGF	Connective Tissue Growth Factor
EC	Endothelial Cell
ECM	Extracellular Matrix
EGFR	Epidermal Growth Factor Receptor
EMT	Epithelial-To-Mesenchymal Transition
ERK	Extracellular Signal-Regulated Kinase
FGF2	Fibroblast Growth Factor 2
FOXO3a	Fork-Head Box O3
Fra-1	Fos-Related Antigen 1
HCC	Hepatocellular Carcinoma
HDF	Human Dermal Fibroblast
HIF	Hypoxia-Inducible Factor
HSC	Hepatic Stellate Cell
Htra3	High-Temperature Requirement A3
ICAM1	Intercellular Adhesion Molecule 1
IDO	Indoleamine 2, 3-Dioxygenase
IGF	Insulin-Like Growth Factor
IGFBP	Insulin-Like Growth Factor Binding Protein
IGF-1R	Insulin-Like Growth Factor Receptor 1
IGFBP-rP	Insulin-like Growth Factor Binding Protein-Related Protein
IL-17RB	Interleukin 17 Receptor B
ImpL2	Ecdysone-Inducible Gene L2
IPF	Idiopathic Pulmonary Fibrosis
JNK	c-Jun N-Terminal Kinase
MACE	Major Adverse Cardiovascular Events
MAPK	Mitogen-Activated Protein Kinase
MEK	Mitogen-Activated Protein Kinase 1
METTL3	Methyltransferase-Like 3
MFAP5	Microfibril-Associated Protein 5
MMP	Matrix Metalloproteinases
MRSS	Modified Rodnan Skin Score
mTOR	Mechanistic Targeting of Rapamycin
MYBBP1A	MYB Binding Protein 1a
NADPH	Nicotinamide Adenine Dinucleotide Phosphate (Reduced)
NAFLD	Non-alcoholic Fatty Liver Disease
NFAT4	Nuclear Factor of Activated T cells 4
NLS	Nuclear Localization Sequence
NO	Nitric Oxide
NOTCH1	Neurogenic Locus Notch Homolog Protein 1
NPC	Nuclear Pore Complex
NYHA	New York Heart Association
PAH	Pulmonary Arterial Hypertension
PAPP-A	Pregnancy-Associated Plasma Protein A
PDAC	Pancreatic Ductal Adenocarcinoma
PD-L1	Programmed Death-Ligand 1
PI3K	Phosphoinositide 3-Kinase
PKM2	Pyruvate Kinase
PPAR-λ	Peroxisome Proliferator-Activated Receptor-λ
ROCK	Rho-Associated Protein Kinase
ROS	Reactive Oxygen Species
RXR-α	Retinoid X Receptor Alpha
SASP	Senescence-Associated Secretory Phenotype
SEMA7A	Semaphorin 7A
SHH	Sonic Hedgehog
SiRNA	Small Interfering Ribonucleic Acid
SMAD	Suppressor of Mothers Against Decapentaplegic
SREBP2	Sterol Regulatory Element-Binding Protein 2
SSc	Systemic Sclerosis
SSc-ILD	Systemic Sclerosis-Associated Interstitial Lung Disease
STAT3	Signal Transducer and Activator of Transcription 3
TAM	Tumor-Associated Macrophages
TCGA	The Cancer Genome Atlas Program
TGF-β	Transforming Growth Factor
TIMP-2	Tissue Inhibitor of Metalloproteinases 2
TLR4	Toll-Like Receptor 4
TME	Tumor Microenvironment
TRα1	Thyroid Hormone Receptor Alpha 1
VDR	Vitamin D Receptor
VEGF	Vascular Endothelial Growth Factor
VEGF-A	Vascular Endothelial Growth Factor-A
VEGFR	Vascular Endothelial Growth Factor receptor
